# Synergistic Anticancer Effects of the 9.2.27PE Immunotoxin and ABT-737 in Melanoma

**DOI:** 10.1371/journal.pone.0024012

**Published:** 2011-09-07

**Authors:** Karianne Risberg, Øystein Fodstad, Yvonne Andersson

**Affiliations:** Department of Tumor Biology, Institute for Cancer Research, Oslo University Hospital Radiumhospitalet, Montebello, Oslo, Norway; Roswell Park Cancer Institute, United States of America

## Abstract

In cancer, combinations of drugs targeting different cellular functions is well accepted to improve tumor control. We studied the effects of a Pseudomonas exotoxin A (PE) - based immunotoxin, the 9.2.27PE, and the BH-3 mimetic compound ABT-737 in a panel of melanoma cell lines. The drug combination resulted in synergistic cytotoxicity, and the cell death observed was associated with apoptosis, as activation of caspase-3, inactivation of Poly (ADP-ribose) polymerase (PARP) and increased DNA fragmentation could be prevented by pre-treatment with caspase and cathepsin inhibitors. We further show that ABT-737 caused endoplasmic reticulum (ER) stress with increased GRP78 and phosphorylated eIF2α protein levels. Moreover, treatment with ABT-737 increased the intracellular calcium levels, an effect which was enhanced by 9.2.27PE, which as a single entity drug had minimal effect on calcium release from the ER. In addition, silencing of Mcl-1 by short hairpin RNA (shRNA) enhanced the intracellular calcium levels and cytotoxicity caused by ABT-737. Notably, the combination of 9.2.27PE and ABT-737 caused growth delay in a human melanoma xenograft mice model, supporting further investigations of this particular drug combination.

## Introduction

Surgical treatment of primary melanoma is associated with high curative rate. However, if the melanoma has progressed to distant metastases, treatment failure is common due to high resistance to current treatment modalities [1], [2]. The median survival rate of metastatic melanoma is 6 months, and less than 5% of the patients survive 5 years, making metastatic melanoma one of the most aggressive cancers in humans [1]. The mitogen-activated protein kinase (MAPK) pathway is constitutively activated in approximately 90% of all melanomas [3], and new drugs targeting this pathway, e.g inhibitors of mutated BRAF or MEK, initially showed promising effects *in vitro*. PLX4032, a selective BRAF^V600E^ inhibitor, caused partial or complete response in early clinical trials [4], [5], however the patients eventually develop resistance. The MEK inhibitors PD0325901 and AZD6244 have also been evaluated in early clinical trials, however, without improvement in the progression-free survival compared to Temozolomide [6], an oral form of the chemotherapeutic drug Dacarbazine used to treat melanoma patients. These results underscore the importance of developing new drugs with yet different mechanisms of action.

An immunotoxin (IT) consists of an antibody linked to a toxin and is designed to specifically kill tumor cells, as the antibody targets cancer cell-associated antigens or antigens over expressed on cancer cells. ITs are taken up through endocytosis, processed within the cell, and cell death is caused by inhibition of protein synthesis through ADP-ribosylation of Elongation Factor 2 and by induction of apoptosis [7]. The 9.2.27PE immunotoxin consists of the Pseudomonas Exotoxin A (PE) linked to the 9.2.27 antibody that targets the high molecular weight-melanoma associated antigen, an antigen expressed on most melanomas and melanoma cell lines [8], [9]. Previous studies show that other PE-based immunotoxins cause strong induction of apoptosis [7], [10]. The 9.2.27PE, on the other hand, caused cell death in melanoma cells primarily through inhibition of protein synthesis, with minor signs of apoptosis [11]. This is possibly due to the high resistance to apoptosis in melanoma cells [2]. Apoptosis is commonly associated with activation of caspases, inactivation of the DNA repair enzyme PARP, chromatin condensation and fragmentation of DNA. It involves two major pathways, the intrinsic/mitochondrial and extrinsic/death receptor apoptotic pathway, which interlace at certain points and ultimately result in cell death [12]. Furthermore, complex interplay between the pro- and anti-apoptotic members of the Bcl-2 family proteins ensures the homeostatic balance within the cell. The Bcl-2 family proteins have an important role in regulating the intrinsic apoptotic pathway, but have also been shown to be involved in maintaining the endoplasmic reticulum (ER) homeostasis, as well as being involved in ER stress signal transduction pathways [13].

The small-molecule inhibitor ABT-737 is developed to neutralize the pro-survival effects of Bcl-2 family proteins. It binds to and inhibits the pro-survival proteins Bcl-2, Bcl-XL and Bcl-W, but not Mcl-1 and A1 [14]. By binding to Bcl-2 and Bcl-XL, pro-apoptotic proteins such as BAX and BAK may be released from Bcl-2 and Bcl-XL and subsequently induce apoptosis as they are proteins of the intrinsic apoptotic pathway. Others have shown that combining ABT-737 with agents decreasing Mcl-1 protein expression act synergistically with ABT-737 [15], [16], and depletion of Mcl-1 restores ABT-737 sensitivity in Fbw7-deficient T-Cell Acute Lymphoblastic Leukemia cells, indicating that elevated Mcl-1 expression is important for Fbw7-deficient cells to evade apoptosis [17]. We have previously shown that by treating melanoma cells with 9.2.27PE, the Mcl-1 protein level is rapidly decreased due to its short half-life [11]. We therefore hypothesized that by combining 9.2.27PE and ABT-737, synergistic cell cytotoxicity could be achieved in melanoma cells with high apoptotic resistance.

We have assessed the effect of combining the 9.2.27PE immunotoxin with the BH-3 mimetic ABT-737 in a panel of melanoma cells. The results suggest that the combination treatment causes strong synergistic cytotoxic effects. Importantly, the combination of the two drugs caused a cytostatic effect on the growth of human melanoma xenograft in mice.

## Materials and Methods

### Immunotoxins and reagents

The immunotoxins 9.2.27PE and 425.3PE, the mAb 9.2.27 and the toxin Pseudomonas Exotoxin A (PE) have previously been described [11]. The agents were diluted in PBS 0.1% human serum albumin (Octapharma, Stockholm, Sweden). Control cells were given 0.1% human serum albumin. ABT-737 and its enantiomer A-793844 (kindly provided by Abbott Laboratories, Abbott Park, IL), were dissolved in DMSO (Sigma-Aldrich, St. Louise, MO), and stored at −20°C until used. For the *in vivo* studies, ABT-737 was dissolved as previously described [18]. The pan-caspase inhibitor Z-VAD-FMK, the cathepsin B/L inhibitor Z-FA-FMK and the caspase-3 inhibitor Z-DEVD-FMK were from Calbiochem (La Jolla, CA). Cycloheximide (CHX) and Staurosporine (STS) were from Sigma-Aldrich, and Tunicamycin was from Sigma Chemical (Castle Hill, Australia). Control cells were given dimethyl sulfoxide (DMSO) (Sigma-Aldrich).

### Antibodies

The following antibodies were used; anti-α-tubulin (Calbiochem, La Jolla, CA), anti-GAPDH (Applied Biosystems, Mulgrave, Australia), anti-PARP (Calbiochem and BD Bioscience, San Jose, CA), anti-caspase-3 (R&D Systems, Minneapolis, MN), anti-BAX, anti-peIF2α, anti-eIF2α (Cell Signaling Technology, La Jolla, CA), anti-Mcl-1, anti-GRP78/BiP and anti-CHOP/GADD 153 (Santa Cruz Biotechnology, Santa Cruz, CA).

### Cell culture

The FEMX, Melmet-1, Melmet-5 and Melmet-44, previously described [19], [20], were kept in RPMI-1640 medium supplemented with 8% heat inactivated fetal calf serum, Hepes and Glutamax (Gibco, Paisley, UK) at 37°C. The MM200 and MelRM (kindly provided by P. Hersey, Calvary Mater Newcastle Hospital, Australia, [21], [22]), were kept in DMEM (Sigma-Aldrich, Castle Hill, Australia) supplemented with 5% fetal calf serum (Sigma-Aldrich), supplemented with 2 mg/ml Sodium Bicarbonate (Chem-Supply, Thermo Scientific, Scoresby, Australia), 20 µg/ml Gentamicin (Pfizer Australia, West Ryde, Australia) at 37°C (100% humidity, 5% CO_2_, 95% air). For all *in vitro* experiments, cells were seeded one day prior to start of the experiments, and the cells were in growth phase and never below 60% confluent at start of treatment. The cells were treated with 100 ng/ml 9.2.27PE or 10 µM ABT-737, unless otherwise indicated. All cell lines were routinely tested and found to be free from contamination with Mycoplasma species.

### Transduction with short hairpin RNA

Mcl-1 were silenced in MelRM cells (MelRMshMcl-1) by transduction using short hairpin RNA (clone ID NM_021960.3-664s1c1, Sigma-Aldrich) according to manufacturer's instructions. Control cells (MelRMshCtr) were generated using non-target control sequence (Product no. SH002, Sigma-Aldrich). The expression level of Mcl-1 was determined by Western Blot.

### Cell viability assay

Cell viability of the FEMX, Melmet-1, Melmet-5, Melmet-44, MelRM and MM200 after treatment for 24 or 48 h with 9.2.27PE (1–1000 ng/ml), ABT-737 (0.1–20 µM) or a combination of 9.2.27PE and ABT-737 was measured using CellTiter 96®AQ_ueous_ One Solution Cell Proliferation Assay (MTS assay; Promega, Madison, WI) as described previously [11] or the VisionBlue™ Quick Cell Viability Assay (BioVision, Mountain View, CA). We rule out that there was a difference between the two cell viability assays, as the cell viability results of FEMX cells treated with 9.2.27PE and ABT-737 were similar using the two different agents. For this cell line, data from both the methods/agents were used to calculate the cell viability results. Cell viability of 9.2.27PE±ABT-737 treated MelRM, MelRMshCtr and MelRMshMcl-1 (12 and 24 h) was determined in a separate experiment.

To determine whether caspases and cathepsins were involved in the decreased cell viability observed, FEMX cells were pre-treated for 45–60 min with Z-VAD-FMK (50 µM)±Z-FA-FMK (50 µM) prior to treatment with 9.2.27PE±ABT-737. Assays were performed in triplicate and repeated at least three times. Furthermore, FEMX and MelRM cells were treated with A-793844, the enantiomer of ABT-737, with concentrations up to 10 µM for 24 and 48 h (48 h: FEMX cells only). Assays were performed in triplicate and repeated three times.

### Caspase activity assay

The activity of caspase-3/7 in FEMX and Melmet-5 cells were measured using the Caspase-Glo 3/7 (Promega, Madison, WI) as described previously [20]. The cells were pre-treated for 45 min with Z-DEVD-FMK (50 µM) prior to treatment with 9.2.27PE±ABT-737, STS (1 µM) or CHX (10 µg/ml). The activity was measured after 24 h according to the manufacturer's instructions. Assays were performed in triplicate and repeated three times.

### Mitochondrial Membrane Potential

The mitochondrial membrane potential (Δψ*m*) in 9.2.27PE±ABT-737 treated FEMX and Melmet-5 cells using the MitoProbe™ JC-1 Assay Kit (Molecular Probes, Eugene, OR) was performed as described previously [11]. In addition FEMX, Melmet-5 and MelRM were treated with ABT-737 for 24, 32 and 48 h. Carbonyl cyanide 3-chlorophenylhydrazone (CCCP) at a final concentration of 50 µM was used as depolarization control. The experiments were repeated three times. MelRM, MelRMshCtr and MelRMshMcl-1 cells were also treated with 9.2.27PE+ABT-737 for 12 h. This experiment was repeated 3 times.

### Western Blot Analysis

FEMX, Melmet-5 and MelRM cells were treated as indicated in the figures and subjected to Western Blot analysis as previously described [11]. Equal amounts of protein (20 µg) were separated by NuPAGE Bis-Tris (Invitrogen, Carlsbad, CA) or SDS-PAGE gels, transferred by electrophoresis to Immobilon membrane (Millipore, Bedford, MA), and probed as previously described [11]. When necessary, the membranes were stripped using Restore™ Western Blot Stripping Buffer (Thermo Scientific Pierce Protein Research Products, Rockford, IL) and re-probed with the desired primary antibody.

### DNA fragmentation Analysis and sub-G_1_ DNA content

TUNEL analysis was performed to determine DNA fragmentation in FEMX and Melmet-5 cells as previously described [11]. The Melmet-5 cells were pre-treated with Z-VAD-FMK (50 µM)+Z-FA-FMK (50 µM) for 45 min. FEMX and Melmet-5 cells were subsequently treated with 9.2.27PE±ABT-737 for 24 h. The experiments were repeated at least three times.

Quantitation of dead cells by measurement of sub-G_1_ DNA content using propidium iodide assay was carried out. FEMX, Melmet-5 and MelRM cells were treated with ABT-737 for 24, 32 and 48 h, harvested, washed with PBS, fixed in icecold MeOH and kept at −20°C until analysis. The cells were washed twice in PBS before subjected to PI (5 µg/ml) and RNase and analyzed on a BDFACSCanto™ (Becton Dickinson, San Jose, CA). The experiment was repeated at least twice for each time point.

### Intracellular calcium levels

FEMX, Melmet-5, MelRM, MelRMshCtr and MelRMshMcl-1 cells were treated with 9.2.27PE±ABT-737 for 12, 16 or 24 h. Cells were collected, washed with PBS, incubated with 1 µM Calcium Green™-1 (Molecular Probes) for 30 min, washed with PBS and subjected to flow cytometric analysis on a Becton Dickinson FACSCanto™ (Becton Dickinson). Intact cells were gated in a forward and side scatter analysis, and results are reported in median fluorescence intensity of the intact cells. Untreated cells were set to the given value 1, and values for the treated cells were calculated accordingly. The experiment was repeated at least three times for each time point per cell line. Analysis was carried out on ∼10.000 cells per sample.

### Ethics Statements

All procedures and experiments involving animals were approved by The National Animal Research Authority and carried out according to the European Convention for the Protection of Vertebrates used for Scientific Purposes. Nude female mice (BALB/c (nu/nu)) were bred in our nude rodent facility. The animals were kept in a specific pathogen-free environment, in positive pressure rooms with filtered and humidified air. The animals were kept under standard conditions, and food and water were supplied *ad libitum*.

### In vivo studies

Nude female mice with Melmet-5 xenografts in the right and left flanks were used. Tumors were size-matched with an average volume of 150–161 mm^3^/group. The animals were divided into four groups; control, 9.2.27PE, ABT-737 and 9.2.27PE+ABT-737. Treatment was initiated with 10–12 tumors per group and mean body weight ≤31 g. ABT-737 (100 mg/kg) was administered once daily on day 1–5 and 15–19 by intraperitoneal injection. The 9.2.27PE immunotoxin (31.25 µg/kg) was given intravenously 5–6 h after ABT-737 treatment on day 1 and 15. All animals administered ABT-737 developed skin rash, no other adverse effects were observed. Two animals in the 9.2.27PE±ABT-737 group developed skin ulcers and were sacrificed on day 8 and 21. For the second experiment, the concentration of ABT-737 was decreased to 50 mg/kg and administered on day 1–5 and 15–19. The 9.2.27PE immunotoxin (31.25 µg/kg) was given 5–6 h after ABT-737 treatment on day 1 and 15. Treatment commenced when the mean tumor volume reached 92–102 mm^3^/group and the mean body weight ≤31 g. For unknown reasons, two mice died shortly after treatment start in the combination group, and were not included in the final analysis. One mice died for unknown reasons on day 15, no other adverse events were recorded. Tumor size was measured 1–3 times weakly by caliper measurements and tumor volume was calculated (tumor volume = L×W^2^/2). Tumor volume was plotted against time (days). Body weight was recorded throughout the experiments. ANOVA was used to calculate statistical differences between groups. Results with a p value less than 0.05 were regarded as significant.

## Results

### 9.2.27PE and ABT-737 reduced cell viability in a panel of melanoma cells

**Figure 1:**
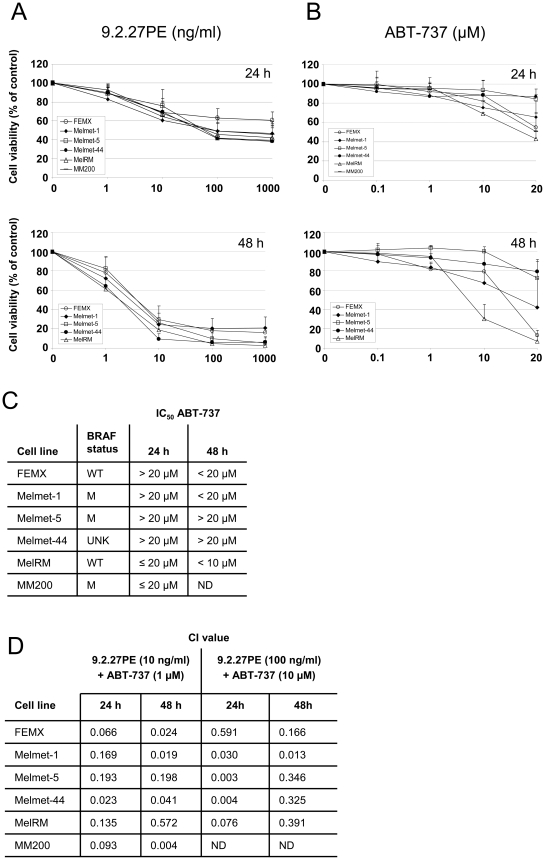
9.2.27PE in combination with ABT-737 causes synergistic cell cytotoxicity in melanoma cells. (A) The 9.2.27PE immunotoxin caused a time- and dose dependent decrease of cell viability in melanoma cells. The effect of the immunotoxin was similar in the different cell lines tested. MM200: 24 h experiment only. (B) ABT-737 caused decreased cell viability in a panel of melanoma cells. The cell cytotoxic effect of ABT-737 differed between the cell lines. (C) B-RAF status and IC_50_ values of ABT-737 treated melanoma cells. WT = wild type, M = BRAF^V600E^, UNK = unknown. (D) The CalcuSyn software program was used to calculate synergistic, additive or antagonistic effects. Combining 10 ng/ml 9.2.27PE+1 µM ABT-737 or 100 ng/ml 9.2.27PE+10 µM ABT-737 caused combination index (CI) values in the 0.59 to 0.003 range indicating synergy to very strong synergy after 24–48 h.

The 9.2.27PE induced a time- and dose dependent decrease in cell viability in the FEMX, Melmet-1, Melmet-5, Melmet-44, MelRM and MM200 cells (Fig. 1A). The effect of 9.2.27PE was consistent in the five cell lines, as opposed to the effect of ABT-737. ABT-737 IC_50_ was obtained in MelRM and MM200 after 24 h (Fig. 1B and C). With increased exposure time to 48 h, IC_50_ was also obtained in FEMX and Melmet-1 cells. Melmet-5 and Melmet-44 cell proved to be less sensitive to ABT-737 with IC_50_>20 µM after 48 h treatment. The sensitivity of the cells to 9.2.27PE or ABT-737 was not related to the BRAF status of these cell lines (Fig. 1C). Furthermore, 24 h treatment with the 425.3PE immunotoxin (100 ng/ml) targeting the epidermal growth factor receptor (EGFR), the antibody 9.2.27 (100 ng/ml) or the PE (100 ng/ml) did not result in decreased cell viability in MelRM cells (data not shown). These results are consistent with results previously obtained for the FEMX cells, emphasizing the specificity of the 9.2.27PE immunotoxin [11].

### 9.2.27PE in combination with ABT-737 synergistically induce apoptosis

To investigate whether the combination of 9.2.27PE and ABT-737 could induce synergistic cytotoxicity in melanoma cells, the cells lines were exposed to different concentrations of the two drugs for 24 and 48 h. The CalcuSyn software program was used to calculate synergistic, additive or antagonistic effects. Combining 10 ng/ml 9.2.27PE+1 µM ABT-737 or 100 ng/ml 9.2.27PE+10 µM ABT-737 caused combination index (CI) values in the 0.59 to 0.003 range, indicating synergy to very strong synergy, after 24–48 h (Fig. 1D and Table S1) [23].

**Figure 2:**
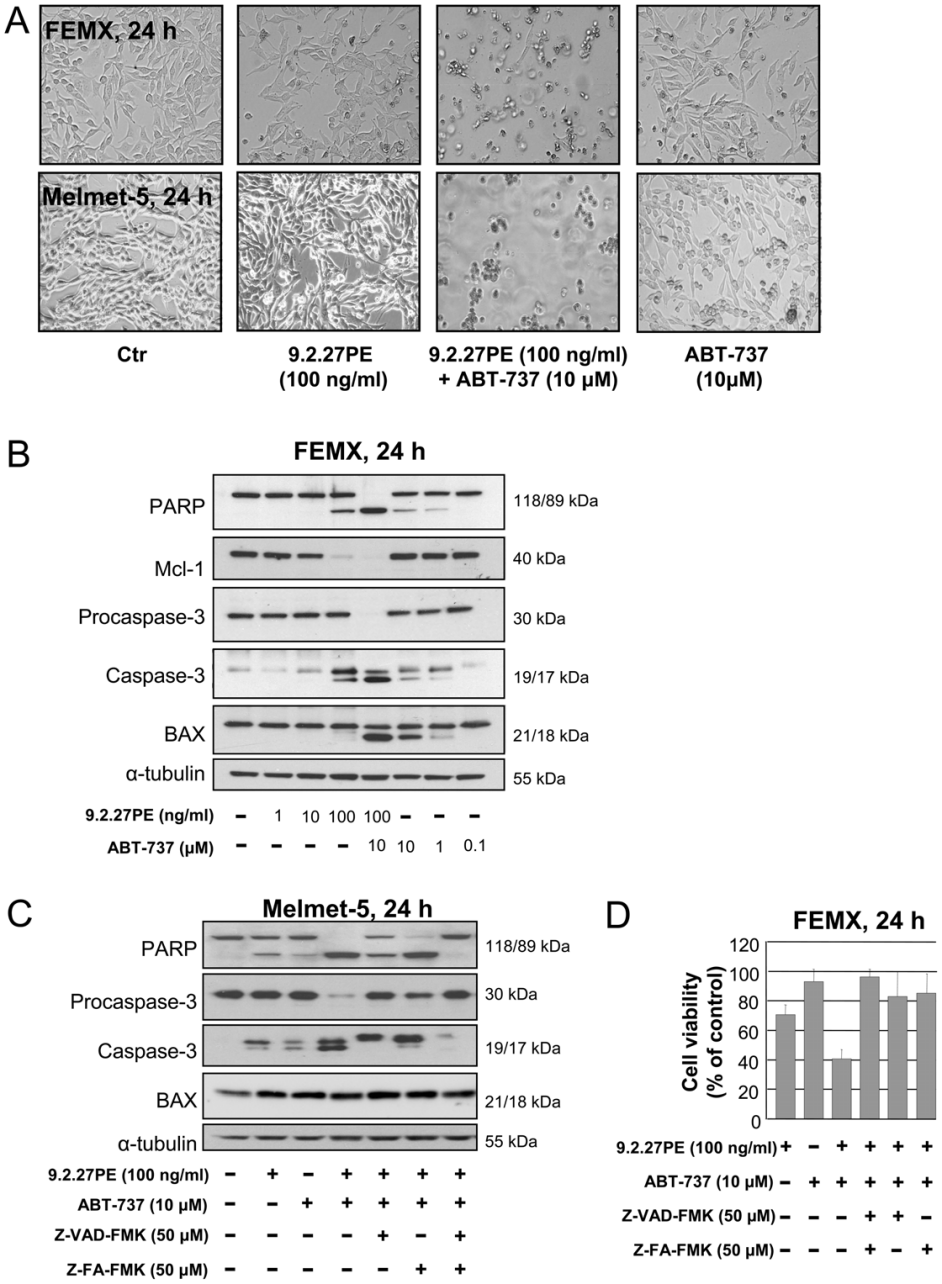
9.2.27PE in combination with ABT-737 induces apoptosis in melanoma cells. (A) FEMX cells and Melmet-5 cells were treated with 9.2.27PE (100 ng/ml)±ABT-737 (10 µM) for 24 h. The combination treatment caused rounding of the cells and detachment from the surface of the flasks as compared to single agent treatment. (B) FEMX cells were treated with various doses of 9.2.27PE (1–100 ng/ml) or various concentrations of ABT-737 (0.1–10 µM) or a combination of 9.2.27PE (100 ng/ml) and ABT-737 (10 µM) for 24 h. Cells were subjected to Western Blot analysis for examination of the levels of PARP, Mcl-1, Caspase-3, BAX and α-tubulin. The blot is a representative of three individual experiments. (C) Melmet-5 cells were treated with 9.2.27PE (100 ng/ml)±ABT-737 (10 µM) for 24 h. Cells were subjected to Western Blot analysis for examination of the levels of PARP, Caspase-3, BAX and α-tubulin. To determine whether caspases and cathepsins were involved in the inactivation of PARP and activation of caspase-3, the cells were pre-treated for 1 h with Z-VAD-FMK (50 µM)±Z-FA-FMK (50 µM) before treatment with 9.2.27PE+ABT-737. The blot is a representative of three independent experiments. (D) Cell viability was measured in FEMX cells treated with 9.2.27PE (100 ng/ml)±ABT-737 (10 µM) for 24 h. To determine whether caspases and cathepsins were involved in the decreased cell viability caused by the combination of 9.2.27PE and ABT-737, the cells were pre-treated with for 1 h with Z-VAD-FMK (50 µM)±Z-FA-FMK (50 µM). The data represents the mean ± SD.

As seen in Figure 2A, 9.2.27PE+ABT-737 caused clear cytotoxic effect in both FEMX and Melmet-5 cells, with morphologically rounding of the cells and detachment from the surface of the flasks, compared to single agent treatments with 9.2.27PE or ABT-737, where only a few cells had started to round up. Similar effects were seen in MelRM cells (data not shown). As both FEMX and MelRM cells proved to be sensitive to the ABT-737 (Fig. 1B), they were further treated with A-793844, the enantiomer and negative control of ABT-737. With concentrations up to 10 µM, no decrease in cell viability was observed after 24 h or 48 h (48 h: FEMX cells only) (Figure S1).

The cytotoxic effect observed when combining 9.2.27PE and ABT-737 was associated with apoptosis, as enhanced PARP inactivation and activation of caspase-3 was observed in both FEMX cells and Melmet-5 cells after 24 h treatment (Fig. 2B and C). Pre-treatment of the Melmet-5 cells with the pan-caspase inhibitor Z-VAD-FMK or the cathepsin B/L inhibitor Z-FA-FMK resulted in a shift upwards of the caspase-3 band from p17 to p19, indicating that the active caspase-3 was blocked (Fig. 2C) [24]. In addition to inhibiting the inactivation of PARP, the combination of the two inhibitors suppressed the processing of the procaspase-3 protein. Furthermore, pre-treatment of the FEMX cells with the combination of Z-VAD-FMK and Z-FA-FMK, inhibited the synergistic cytotoxic effect caused by 9.2.27PE (100 ng/ml)+ABT-737 (10 µM) and 9.2.27PE (10 ng/ml)+ABT-737 (1 µM) in FEMX cells (Fig. 2D and data not shown), combinations which caused synergistic cytotoxic effects (Fig. 1D).

A caspase-3/7 activity assay was utilized to confirm activity of the cleaved caspase-3 band detected on Western Blot. As seen in Figure S2, 9.2.27PE±ABT-737 increased caspase-3/7 activity after 24 h in both the FEMX and the Melmet-5 cells. The caspase-3/7 acticity was effectively blocked by pre-treatment with the caspase-3 II inhibitor (Z-DEVD-FMK, 50 µM). STS (1 µM), which we previously have shown to induce caspase-3/7 activity in FEMX cells [11], was used as a positive control, and CHX, not causing caspase-3/7 activity [25], was used as a negative control.

### 9.2.27PE in combination with ABT-737 cause strong depolarization of the mitochondrial membrane

**Figure 3:**
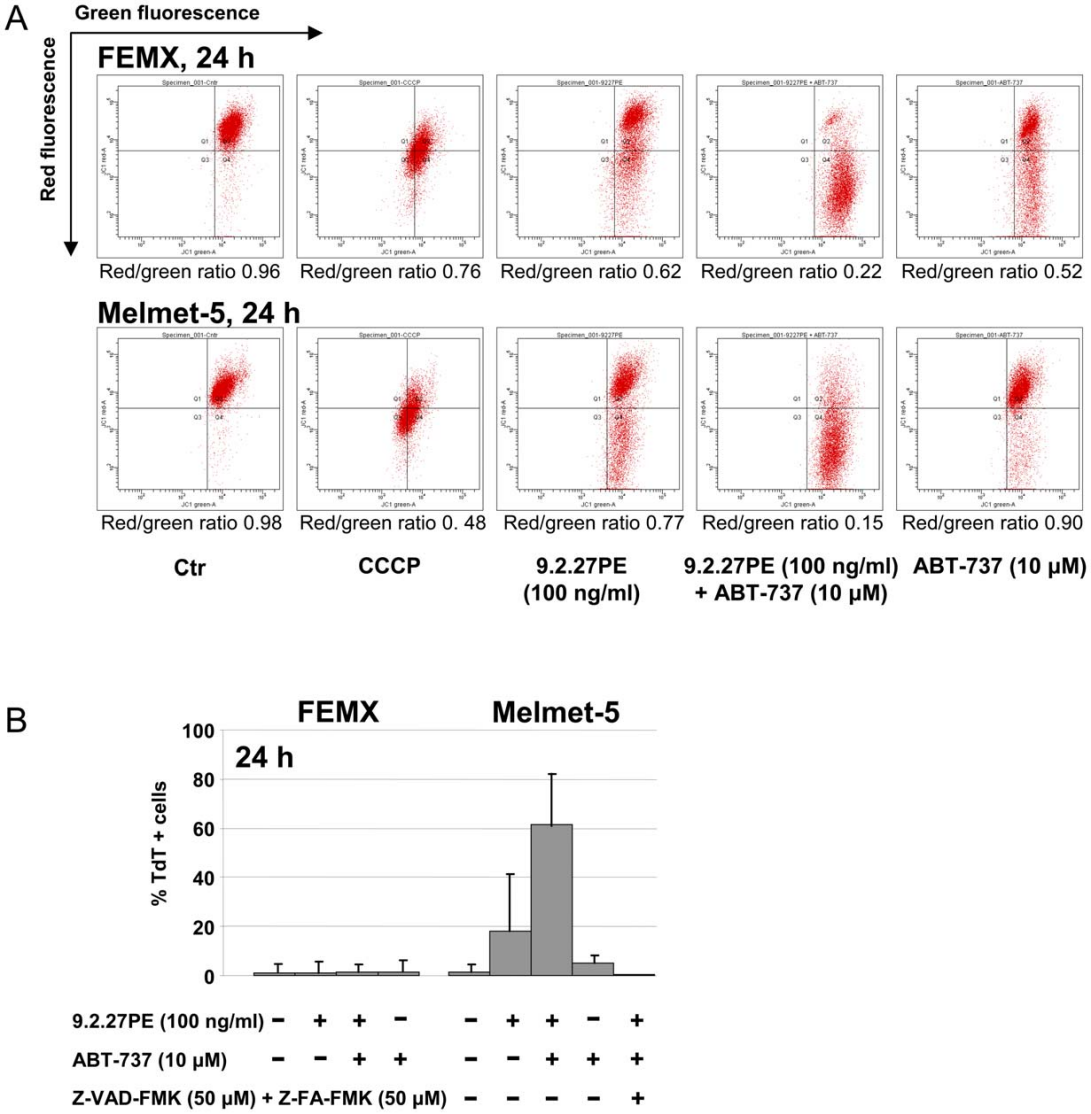
9.2.27PE in combination with ABT-737 induces strong depolarization of the mitochondrial membrane. (A) FEMX and Melmet-5 cells were treated with 9.2.27PE (100 ng/ml)±ABT-737 (10 µM) for 24 h and subsequently stained with JC-1, an indicator for mitochondrial membrane potential. A decrease in the red/green fluorescence ratio indicates decreased mitochondrial membrane potential. CCCP was used as a depolarization control. The figures shown are examples from one representative experiment. The red/green ratio value is based on three independent experiments. (B) DNA fragmentation was analyzed by TUNEL assay. FEMX and Melmet-5 cells were treated with 9.2.27PE (100 ng/ml)±ABT-737 (10 µM) for 24 h. In addition, the Melmet-5 cells were pre-treated with for 1 h with Z-VAD-FMK (50 µM)±Z-FA-FMK (50 µM) to determine whether caspases and cathepsins were involved in the DNA fragmentation observed in this cell line.

Decreased Δψ*m* is linked to apoptosis as pro-apoptotic proteins, which are released from the mitochondria into the cytosol, are capable of activating caspase-3 and inducing DNA fragmentation. We used the cation dye JC-1 to detect whether the Δψ*m* was affected by 9.2.27PE±ABT-737 in FEMX and Melmet-5 cells. JC-1 fluoresces red in intact mitochondria, and upon collapse of the membrane, JC-1 accumulates in the cytoplasm where it fluoresces green. A decrease in red/green ratio indicates depolarization of the mitochondrial membrane. In FEMX cells, the red/green ratio decreased from 0.62 and 0.52 for the 9.2.27PE and ABT-737 treatments, respectively, to 0.22 for the combination treatment (Fig. 3A). Similar results were obtained for the Melmet-5 cells, where the red/green ratio decreased from 0.77 and 0.90 for the 9.2.27PE and ABT-737 treatments, respectively, to 0.15 for the 9.2.27PE+ABT-737 combination. CCCP was used as a positive control for depolarization.

We next analysed DNA fragmentation by TUNEL assay. As seen in Figure 3B, 24 h treatment with 9.2.27PE and ABT-737 caused DNA fragmentation in ∼20% and 5% of the Melmet-5 cells, respectively. Combining the two drugs, the percentage of DNA fragmentation increased to ∼60%. Pre-incubation with Z-VAD-FMK+Z-FA-FMK inhibited the DNA fragmentation, indicating that caspases and cathepsins are involved in the cell death process. For the combination treatment, the caspase-3/7 activation in Melmet-5 cells (Figure S2) did not directly correlate with the strong increase in DNA fragmentation (Fig. 3B) suggesting that other factors than caspase-3/7 might be involved in DNA fragmentation [26]. In a previous study with 9.2.27PE treated FEMX cells, no DNA fragmentation was detected using the TUNEL assay [11]. In the present study, no DNA fragmentation was detected in FEMX cells treated with 9.2.27PE±ABT-737 (Fig. 3B). As the combination treatment caused strong depolarization of the mitochondrial membrane in addition to caspase-3 activity, we conclude that under the above mentioned conditions, and after treatment with STS [11], DNA fragmentation does not take place in the FEMX cells.

### ABT-737 induces ER stress and cell death

**Figure 4:**
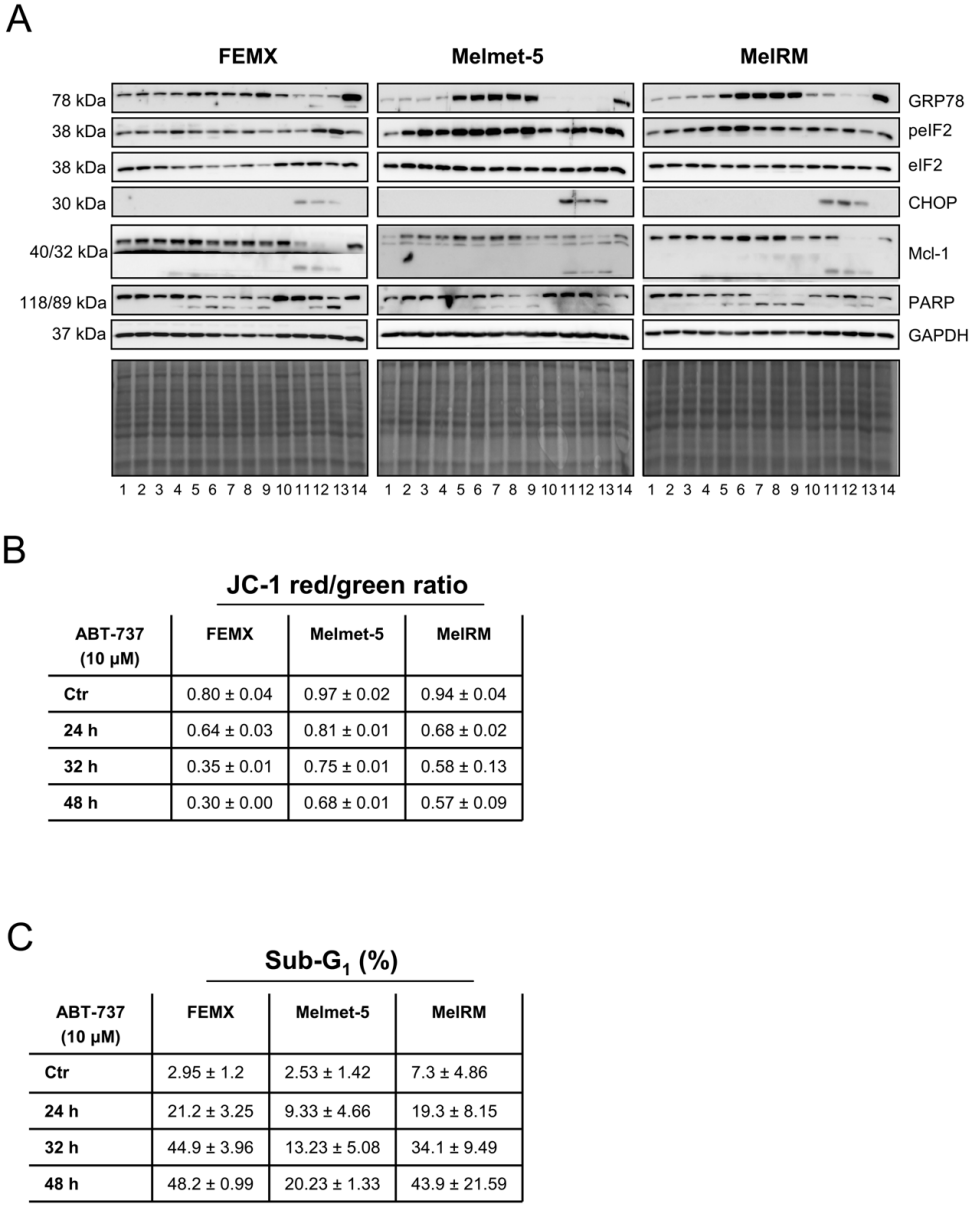
ABT-737 induces ER stress and cell death in melanoma cells. (A) Increased GRP78 and peIF2α protein levels in FEMX, Melmet-5 and MelRM melanoma cells treated with ABT-737 (10 µM) up to 48 h, indicating ER stress. CHOP protein was only increased with 9.2.27PE treatment. Both ABT-737 and 9.2.27PE caused inactivation of PARP, indicating apoptosis. GAPDH and amidoblack staining was used as a loading control. Tunicamycin (3 µM), was used as a positive control for ER stress. The blot is representative of at least three independent experiments, except for the 32 and 48 h time point where the experiments were repeated twice. Lane 1: Vehicle (DMSO), 2: ABT-737 1 h, 3: ABT-737 2 h, 4: ABT-737 4 h, 5: ABT-737 8 h, 6: ABT-737 16 h, 7: ABT-737 24 h, 8: ABT-737 32 h 9: ABT-737 48 h, 10: Vehicle (PBS 0.1% human serum albumin), 11: 9.2.27PE 8 h, 12: 9.2.27PE 16 h, 13: 9.2.27PE 24 h, 14: Tunicamycin 8 h. (B) Mitochondrial membrane potential was measured using the JC-1 dye. A decrease in red/green ratio is indicative of depolarization of the mitochondrial membrane. The data represents the mean ± SD. (C) FEMX, Melmet-5 and MelRM cells were treated with ABT-737 (10 µM) for 24–48 h before stained with propidium iodide for quantitation of dead cells. An increase in the percentage sub-G1 represents increased cell death compared to control cells. The data represents the mean ± SD.

We wanted to investigate which mechanisms could contribute to the synergistic cell death effect observed by the 9.2.27PE+ABT-737 combination treatment. As prolonged ER stress can lead to apoptosis and autophagy [13], we first investigated whether ABT-737 was able to induce ER stress in melanoma cells. The commonly used markers for ER stress, GRP78 and peIF2α, were increased in the ABT-737 treated FEMX, Melmet-5 and MelRM cells (Fig. 4A), the increase was more pronounced in the Melmet-5 and MelRM cells. Treatment with 9.2.27PE did not induce increased protein levels of GRP78, however an increase in peIF2α was observed. Tunicamycin, which is known to induce ER stress, was used as a positive control for increased GRP78. During ER stress, GRP78 dissociates from the three ER membrane proteins PERK, IRE1 and ATF6. This process leads to activation of these proteins, transcriptional activation of *CHOP* and may result in apoptosis [27]. CHOP was induced upon 9.2.27PE treatment, but interestingly not upon ABT-737 treatment in FEMX, Melmet-5 or MelRM cells despite increased levels of GRP78 and peIF2α. Further investigations are needed to clarify the involvement of CHOP in 9.2.27PE and ABT-737 treated melanoma cells. Nevertheless, ABT-737 treatment caused decreased Δψ*m* (Fig. 4B), inactivation of PARP (Fig. 4A) and increased sub-G1 fraction (Fig. 4C), after 48 h in FEMX, Melmet-5 and MelRM cells. These results indicate that ABT-737 was able to induce ER stress and cell death in melanoma cells *in vitro*.

### 9.2.27PE enhances Ca^2+^ efflux from Endoplasmic reticulum induced by ABT-737

**Figure 5:**
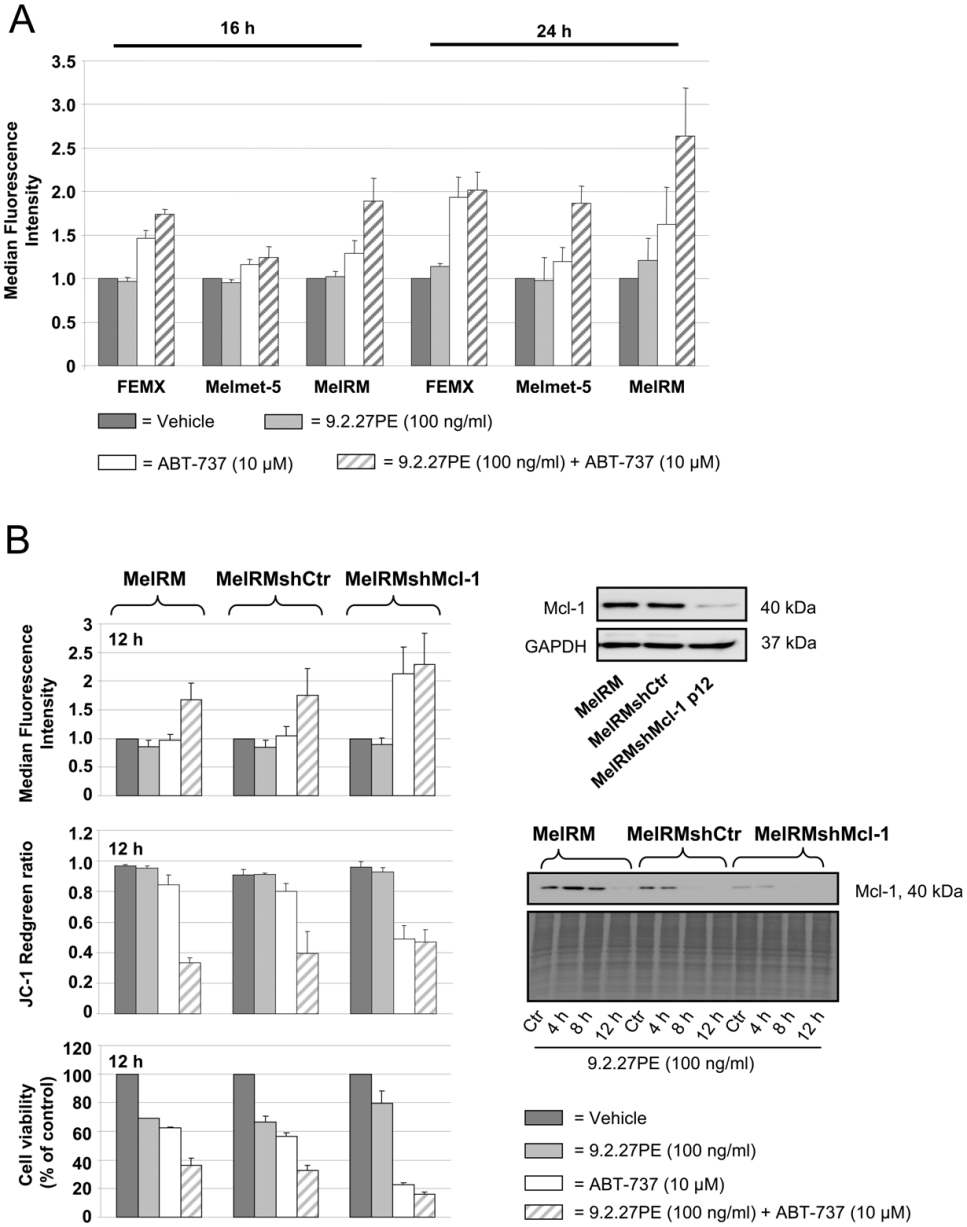
Calcium release caused by 9.2.27PE±ABT-737 in melanoma cells. (A) FEMX, Melmet-5 and MelRM cells were subjected to 9.2.27PE (100 ng/ml)+ABT-737 (10 µM) for 16 and 24 h before subjected to flow cytometric analysis using Calcium Green™-1. The 9.2.27PE enhanced calcium release caused by ABT-737. The data shown are the mean ± SD for at least three independent experiments. (B) MelRM, MelRMshCtr and MelRMshMcl-1 were subjected to 9.2.27PE (100 ng/ml)+ABT-737 (10 µM). [Ca^2+^]_i_ levels, mitochondrial membrane potential and cell viability was measured after 12 h. Knockdown of Mcl-1 using shRNA enhanced the calcium release and decreased cell viability caused by ABT-737, an effect which could not be enhanced by 9.2.27PE. The data shown are the mean ± SD. P12 = passage 12, the highest passage used for this experiment.

One function of the ER is storage and regulation of calcium. We therefore investigated whether 9.2.27PE±ABT-737 caused calcium release from the ER in melanoma cells. ABT-737 caused an increase in intracellular calcium ([Ca^2+^]_i_) levels in FEMX, Melmet-5 and MelRM cells after 16 and 24 h (Fig. 5A). The increased [Ca^2+^]_i_ levels was most pronounced in FEMX cells. The 9.2.27PE, which as a single agent had minor effect on calcium release, enhanced the [Ca^2+^]_i_ levels caused by ABT-737. The enantiomer A-793844 was not able to elicit increased [Ca^2+^]_i_ levels in Melmet-5 or MelRM cells after 16 and 24 h (data not shown). FEMX cells were not tested with the A-723844 for this purpose.

In contrast to the control cells (MelRM and MelRMshCtr), silencing of Mcl-1 in the MelRM (MelRMshMcl-1) lead to significantly increased [Ca^2+^]_i_ levels, decreased Δψ*m* and decreased cell viability after 12 h ABT-737 treatment, effects which could not be further enhanced by 9.2.27PE (Fig. 5B). As the Mcl-1 level after 12 h 9.2.27PE treatment was undetectable (Fig. 5B) in all three cell lines, these results indicate that silencing of Mcl-1 through shRNA or 9.2.27PE treatment is responsible for the increased [Ca^2+^]_i_ levels during ABT-737 treatment. The control cells, MelRM and MelRMshCtr, showed only a significant increase in [Ca^2+^]_i_ levels when treated with 9.2.27PE+ABT-737, ant not with ABT-737 as a single agent treatment. The 9.2.27PE had no effect on calcium release in either of the MelRM cell lines after 12 h. Interestingly, 9.2.27PE caused less decrease of cell viability in MelRMshMcl-1 cells compared to control cells after 12 h (Fig. 5B) and 24 h (Figure S3), indicating a role of Mcl-1 in mediating the cytotoxicity of 9.2.27PE.

### 9.2.27PE and ABT-737 reduces tumor growth *in vivo*

**Figure 6:**
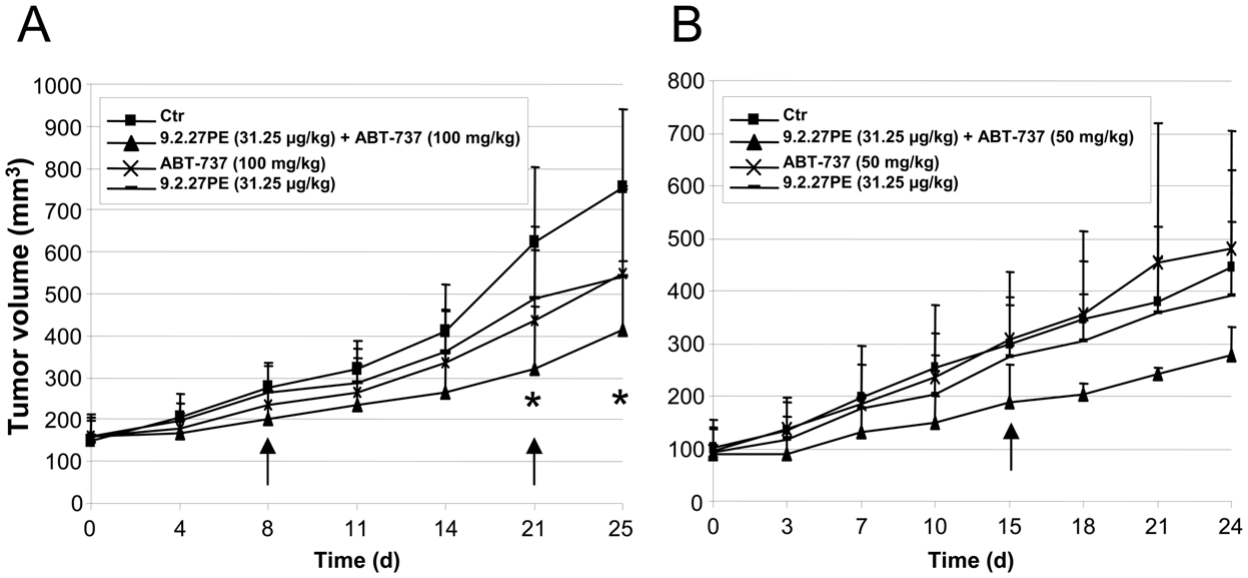
Effect of 9.2.27PE in combination with ABT-737 in a Melmet-5 xenograft model. (A) Nude mice were treated with ABT-737 (100 mg/kg) day 1–5 and 15–19 and 9.2.27PE (31.25 g/kg) on day 1 and 15. (B) Nude mice were treated with ABT-737 (50 mg/kg) day 1–5 and 15–19 and 9.2.27PE (31.25 g/kg) on day 1 and 15. Tumor size was measured 1–3 times weakly by caliper measurements and tumor volume was calculated (tumor volume = L×W^2^/2). * indicates p value<0.05, using ANOVA. Arrows indicate the day animals in the combination group were sacrificed due to adverse events (skin ulcers).

Our *in vitro* data demonstrated that a combination of 9.2.27PE and ABT-737 caused synergistic cytotoxic effects in a panel of melanoma cells. To determine whether these effects could be validated *in vivo*, nude mice with subcutaneous Melmet-5 xenografts were treated with 9.2.27PE and ABT-737. As seen in Figure 6A, the combination regime of 9.2.27PE (31.25 µg/kg)+ABT-737 (100 mg/kg) caused significant cytostatic effect after 21 and 25 d (p<0.05). As all animals receiving 100 mg/kg ABT-737 developed skin rash, the concentration of ABT-737 was halved in the second experiment (Fig. 6B). Skin rash was not observed, but the combination treatment did not significantly decrease inhibition of tumor growth, possibly due to large tumor volume variations within the groups. There was, however, a tendency to stronger cytostatic effect caused by the combination treatment compared to treatment with ABT-737 or 9.2.27PE as single agents. Body weight was stable throughout the experiments (Figure S4).

## Discussion

Cancer cells are characterized by uncontrolled proliferation and apoptotic resistance. Melanoma cells are particularly drug resistant, and drug-resistance is often correlated with apoptosis evasion [2]. Drug resistance is likely a reason for treatment failure in melanoma patients. The Bcl-2 family proteins may serve as an important and widely applicable target for anti-cancer immunotherapeutic strategies, and spontaneous cellular immune responses against the Bcl-2 family proteins have been identified as frequent features in cancer patients [28]. These observations underscore that the Bcl-2 family proteins are natural targets for the immune system, hence, these proteins may serve as an important target for anti-cancer immunotherapeutic strategies [28]. The combined use of several drugs targeting different cellular functions, e.g. the Bcl-2 family, is therefore one approach to achieve tumor control in cancer, including melanoma.

In the present study we show that the melanoma specific immunotoxin 9.2.27PE in combination with the BH-3 mimetic ABT-737, caused strong synergistic cytotoxic effect in the panel of melanoma cells, independently of their BRAF status and, as previously reported [20], their sensitivity to Dacarbazine. Depolarisation of the mitochondrial membrane, activation of caspase-3, inactivation of PARP and increased DNA fragmentation was obtained using the combination treatment, hence, cell death involved induction of the intrinsic apoptotic pathway.

To elucidate which mechanisms are responsible for the synergistic cytotoxic effect observed when treating melanoma cells with 9.2.27PE+ABT-737, we looked to the ER, as ABT-737 has been reported to induce the transcription factor ATF4 in other types of cancer [29], [30]. Disturbances in the normal functions of the ER lead to unfolded protein response and ER stress [31]. The initial intent of the unfolded protein response is to adapt to these changes and re-establish normal ER function. This happens through dissociation of GRP78 from the three ER chaperones PERK, IRE1 and ATF6, resulting in increased protein levels of GRP78 and activation of the three ER stress pathways (PERK–eIF2α–ATF4, ATF6-ATF6 splicing, and IRE1-XBP1) [31], [32]. Unresolved and prolonged ER stress will eventually result in apoptosis [32]. In this study, ABT-737 treatment resulted in increased protein levels of GRP78 and peIF2α, indicative of ER stress. In addition, ABT-737 treatment resulted in decreased Δψ*m*, inactivation of PARP and increased sub-G1 population, mechanisms related to apoptosis. These results indicate that ABT-737 as a single agent induces ER stress which ultimately results in cell death.

Another function of the ER is storage and regulation of calcium. Upon ER stress, calcium is released from the ER into the cytosol which can be taken up by juxtaposed mitochondria and result in decreased Δψ*m* [33]. In this study we show that ABT-737 increased [Ca^2+^]_i_ levels, and that 9.2.27PE, which as a single agent had minimal effect on calcium release, enhanced the ABT-737 induced calcium release from ER. This result suggests that 9.2.27PE directly or indirectly has an effect on ER and is effective for sensitizing the melanoma cells to ABT-737. Others have shown that decreased Mcl-1 expression is effective for sensitizing cancer cells to ABT-737 and induce apoptosis [15], [16]. As 9.2.27PE lead to rapid decrease in Mcl-1 protein levels [11], this lead us to investigate whether knockdown of Mcl-1 protein expression could increase the [Ca^2+^]_i_ levels, decrease the Δψ*m* and decrease the cell viability in ABT-737 treated melanoma cells. In contrast to the control cells (MelRM and MelRMshCtr), ABT-737 treated MelRMshMcl-1 cells showed significantly increased [Ca^2+^]_i_ levels, decreased Δψ*m* and decreased cell viability, effects which could not be further enhanced by 9.2.27PE in the MelRMshMcl-1 cells. These results indicate that Mcl-1 has an inhibitory effect on calcium release from the ER, and that strongly decreased Mcl-1 protein levels in melanoma cells after treatment with 9.2.27PE (Fig. 4A) might be responsible for enhanced [Ca^2+^]_i_ levels in ABT-737 treated cells. Calcium release has been shown to take place through the inositol 1,4,5-triphosphate receptor and the ryanodine receptors in the ER membrane [13], [31]. Bcl-2 family proteins are involved in this process, as several members, including Mcl-1, have been shown to interact with e.g. inositol 1,4,5-triphosphate receptor [34]–[38]. The precise mechanisms how the 9.2.27PE+ABT-737 drug combination causes calcium release and synergistic cell death in melanoma cells is under investigation.

The downstream effectors of increased [Ca^2+^]_i_ levels, are numerous and thoroughly reviewed by Xu et al [31]. Of importance to the present study is the mitochondrial uptake of excessive Ca^2+^ leading to decreased Δψ*m*, activation of the intrinsic apoptotic pathway via release of a number of pro-apoptotic factors including cytochrome *c*, apoptosis inducing factor (AIF) and Smac/Diablo [39]. The enhanced [Ca^2+^]_i_ levels caused by 9.2.27PE+ABT-737 treatment could therefore explain the increased mitochondrial membrane depolarization caused by the combination treatment compared to single agent treatments, an effect that subsequently resulted in synergistic decrease in cell viability and strong induction of apoptosis.

As ABT-737 leads to liver deterioration in Mcl-1 knockout mice, inhibition of Mcl-1 should be done in a tumor specific manner to induce apoptosis and tumor specific growth control [40]. We have previously shown that Mcl-1 protein levels rapidly is decreased in cancer cells when targeted with several of our Pseudomonas Exotoxin A based immunotoxins [7], [11], of which one (the MOC31PE targeting the EpCAM antigen) is currently in clinical trial phase I/II (www.clinicaltrials.gov, assessed July 20, 2011). Furthermore, ABT-263/Navitoclax, the oral form of ABT-737, is currently in phase I and phase II clinical trials (www.clincaltrials.gov, assessed July 20, 2011). From a clinical perspective, 9.2.27PE+ABT-263 is therefore an interesting treatment combination for further investigations. Importantly, administration of 9.2.27PE+ABT-737 caused growth delay effect in Melmet-5 xenograft tumors, implying that this particular drug combination show the ability to kill melanoma cells *in vivo*. However, as only tumor growth delay was obtained, treatment of solid melanoma tumors might prove to be challenging. Immunotoxins and ABT-737 as single agents have, nevertheless, been shown to be effective in hematological tumors, where in fact one immunoconjugate consisting of an IL-2 fused to a modified Diphteria toxin (Ontak®) is approved by the Food and Drug Administration is an option for patient with cutaneous T-cell lymphoma. To circumvent the challenges related to treatment of solid metastatic melanoma tumors, targeting circulating melanoma cells or minimal residual disease which is present in patients treated with e.g. the new BRAF or MEK inhibitors (as these patients do relapse after some time), could be a viable option. As melanoma is a diverse disease, and the combination treatment with 9.2.27PE+ABT-737 was able to efficiently cause cell death in several melanoma cell lines tested *in vitro*, this approach could reach a broader patient group, not limited by specific mutations.

Taken together, the combination of 9.2.27PE and the ABT-737 caused synergistic cell death in a panel of melanoma cells, and the cell death observed was associated with apoptosis involving the intrinsic apoptosis pathway. ER stress caused by ABT-737, and enhanced Ca^2+^ release caused by ABT-737 as a single agent or the 9.2.27PE+ABT-737 combination treatment, likely contributed to the cell cytotoxicity and the synergistic anti-melanoma activity. Importantly, cytostatic effect was obtained in a melanoma xenograft model, indicating that this combination is effective *in vivo*. Whether melanoma cells *in vivo* will recruit additional survival mechanisms after treatment with the oral form of ABT-737, the ABT-263, resulting in resistance to the drug, remains to be investigated. Lymphoma cells *in vitro* acquired resistance after long-term exposure ABT-737 due to up-regulation of Mcl-1 and A1 [41]. We do, however, know that long term exposure of low doses of 9.2.27PE causes cell cytotoxicity to all melanoma cells *in vitro*, due to the general inhibition of protein synthesis leading to cell death (data not published). Generation of immunotoxin-resistant cells is therefore not likely to occur.

## Supporting Information

Figure S1 The effect of A-793844 on cell viability in melanoma cells. FEMX and MelRM cells were treated with the enantiomer A-793844 (the negative control of ABT-737) for the indicated time points. No significant decrease in cell viability was observed. The data represents the mean ± SD.
(TIF)

Figure S2 Both 9.2.27PE and ABT-737 causes active caspase-3 in melanoma cells. FEMX and Melmet-5 cells were treated with 9.2.27PE and ABT-737 as indicated. Active caspase-3 was measured using the Caspase-Glo 3/7 from Promega. Caspase-3 activation could be inhibited by 45 min pre-incubation with the caspase-3 inhibitor Z-DEVD-FMK. Staurosporine (STS) was used as a positive control for caspase-3 activation, and CHX was used as a negative control.
(TIF)

Figure S3 Calcium release caused by 9.2.27PE±ABT-737 in melanoma cells. MelRM, MelRMshCtr and MelRMshMcl-1 were subjected to 9.2.27PE (100 ng/ml)+ABT-737 (10 µM). [Ca^2+^]_i_ levels, and cell viability was measured after 24 h. Knockdown of Mcl-1 using shRNA enhanced the calcium release and decreased cell viability caused by ABT-737, an effect which could not be enhanced by 9.2.27PE. The data shown are the mean ± SD. p9 = passage 9, the highest passage used for this experiment.
(TIF)

Figure S4 Body weight of nude mice treated with 9.2.27PE±ABT-737. Nude mice were treated with ABT-737 (100 mg/kg) day 1–5 and 15–19 and 9.2.27PE (31.25 µg/kg) on day 1 and 15 (left panel) or ABT-737 (50 mg/kg) day 1–5 and 15–19 and 9.2.27PE (31.25 µg/kg) on day 1 and 15 (right panel). Body weight was measured throughout the experiment.
(TIF)

Table S1 9.2.27PE in combination with ABT-737 causes synergistic cell cytotoxicity in melanoma cells. Fractional inhibition = fraction decreased cell viability after treatment, control cells were set to “1”, CI = combination index. CI values below 0.9 indicate synergistic effect. ND = not done.
(DOC)
