# Supplementary material for: Synergistic Anticancer Effects of the 9.2.27PE Immunotoxin and ABT-737 in Melanoma
Source: PLoS One. 2011 Sep 7;6(9):e24012. doi: 10.1371/journal.pone.0024012 (PMC3168478; doi:10.1371/journal.pone.0024012)
Supplement: Table S1 — 9.2.27PE in combination with ABT-737 causes synergistic cell cytotoxicity in melanoma cells. Fractional inhibition = fraction decreased cell viability after treatment, control cells were set to “1”, CI = combination index. CI values below 0.9 indicate synergistic effect. ND = not done. (DOC) [file pone.0024012.s005.doc]

| **FEMX**  Supplementary Table 1 | | | | | |
| --- | --- | --- | --- | --- | --- |
| Drug | | 24 h | | 48 h | |
| 9.2.27PE (ng/ml) | ABT-737 (μM) | Fractional inhibition | CI | Fractional inhibition | CI |
|  |  |  |  |  |  |
| (D)_1_ |  |  |  |  |  |
| 1 |  | 0.09 |  | 0.22 |  |
| 10 |  | 0.28 |  | 0.70 |  |
| 100 |  | 0.33 |  | 0.82 |  |
| 1000 |  | 0.36 |  | 0.84 |  |
|  |  |  |  |  |  |
|  | (D)_2_ |  |  |  |  |
|  | 0.1 | 0.0001 |  | 0.03 |  |
|  | 1 | 0.07 |  | 0.18 |  |
|  | 10 | 0.05 |  | 0.21 |  |
|  | 20 | 0.40 |  | 0.86 |  |
|  |  |  |  |  |  |
| (D)_1_ + (D)_2_ |  |  |  |  |  |
| 1 | 0.1 | 0.17 | 0.031 | 0.30 | 0.246 |
| 10 | 1 | 0.59 | 0.066 | 0.91 | 0.024 |
| 100 | 10 | 0.66 | 0.591 | 0.93 | 0.166 |

| **Melmet-1** | | | | | |
| --- | --- | --- | --- | --- | --- |
| Drug | | 24 h | | 48 h | |
| 9.2.27PE (ng/ml) | ABT-737 (μM) | Fractional inhibition | CI | Fractional inhibition | CI |
|  |  |  |  |  |  |
| (D)_1_ |  |  |  |  |  |
| 1 |  | 0.173 |  | 0.277 |  |
| 10 |  | 0.393 |  | 0.757 |  |
| 100 |  | 0.510 |  | 0.793 |  |
| 1000 |  | 0.533 |  | 0.790 |  |
|  |  |  |  |  |  |
|  | (D)_2_ |  |  |  |  |
|  | 0.1 | 0.04 |  | 0.103 |  |
|  | 1 | 0.12 |  | 0.163 |  |
|  | 10 | 0.247 |  | 0.327 |  |
|  | 20 | 0.347 |  | 0.577 |  |
|  |  |  |  |  |  |
| (D)_1_ + (D)_2_ |  |  |  |  |  |
| 1 | 0.1 | 0.193 | 1.847 | 0.29 | 6.195 |
| 10 | 1 | 0.433 | 0.169 | 0.83 | 0.019 |
| 100 | 10 | 0.707 | 0.030 | 0.92 | 0.013 |

| **Melmet-5** | | | | | |
| --- | --- | --- | --- | --- | --- |
| Drug | | 24 h | | 48 h | |
| 9.2.27PE (ng/ml) | ABT-737 (μM) | Fractional inhibition | CI | Fractional inhibition | CI |
|  |  |  |  |  |  |
| (D)_1_ |  |  |  |  |  |
| 1 |  | 0.11 |  | 0.17 |  |
| 10 |  | 0.24 |  | 0.74 |  |
| 100 |  | 0.58 |  | 0.90 |  |
| 1000 |  | 0.61 |  | 0.94 |  |
|  |  |  |  |  |  |
|  | (D)_2_ |  |  |  |  |
|  | 0.1 | 0.047 |  | 0.0001 |  |
|  | 1 | 0.047 |  | 0.0002 |  |
|  | 10 | 0.063 |  | 0.0003 |  |
|  | 20 | 0.157 |  | 0.27 |  |
|  |  |  |  |  |  |
| (D)_1_ + (D)_2_ |  |  |  |  |  |
| 1 | 0.1 | 0.13 | 0.812 | 0.17 | 2.230 |
| 10 | 1 | 0.40 | 0.193 | 0.81 | 0.198 |
| 100 | 10 | 0.89 | 0.003 | 0.93 | 0.346 |

| **Melmet-44** | | | | | |
| --- | --- | --- | --- | --- | --- |
| Drug | | 24 h | | 48 h | |
| 9.2.27PE (ng/ml) | ABT-737 (μM) | Fractional inhibition | CI | Fractional inhibition | CI |
|  |  |  |  |  |  |
| (D)_1_ |  |  |  |  |  |
| 1 |  | 0.103 |  | 0.357 |  |
| 10 |  | 0.303 |  | 0.907 |  |
| 100 |  | 0.587 |  | 0.940 |  |
| 1000 |  | 0.617 |  | 0.937 |  |
|  |  |  |  |  |  |
|  | (D)_2_ |  |  |  |  |
|  | 0.1 | 0.080 |  | 0.03 |  |
|  | 1 | 0.130 |  | 0.063 |  |
|  | 10 | 0.117 |  | 0.127 |  |
|  | 20 | 0.133 |  | 0.207 |  |
|  |  |  |  |  |  |
| (D)_1_ + (D)_2_ |  |  |  |  |  |
| 1 | 0.1 | 0.18 | 0.371 | 0.367 | 5.678 |
| 10 | 1 | 0.623 | 0.023 | 0.937 | 0.041 |
| 100 | 10 | 0.887 | 0.004 | 0.943 | 0.325 |

| **MelRM** | | | | | |
| --- | --- | --- | --- | --- | --- |
| Drug | | 24 h | | 48 h | |
| 9.2.27PE (ng/ml) | ABT-737 (μM) | Fractional inhibition | CI | Fractional inhibition | CI |
|  |  |  |  |  |  |
| (D)_1_ |  |  |  |  |  |
| 1 |  | 0.07 |  | 0.39 |  |
| 10 |  | 0.32 |  | 0.81 |  |
| 100 |  | 0.54 |  | 0.95 |  |
| 1000 |  | 0.58 |  |  |  |
|  |  |  |  |  |  |
|  | (D)_2_ |  |  |  |  |
|  | 0.1 | 0.01 |  | 0.02 |  |
|  | 1 | 0.03 |  | 0.05 |  |
|  | 10 | 0.31 |  | 0.70 |  |
|  | 20 | 0.57 |  | 0.93 |  |
|  |  |  |  |  |  |
| (D)_1_ + (D)_2_ |  |  |  |  |  |
| 1 | 0.1 | 0.131 | 0.601 | 0.304 | 1.903 |
| 10 | 1 | 0.468 | 0.135 | 0.866 | 0.572 |
| 100 | 10 | 0.855 | 0.076 | 0.983 | 0.391 |

| **MM200** | | | | | |
| --- | --- | --- | --- | --- | --- |
| Drug | | 24 h | | 48 h | |
| 9.2.27PE (ng/ml) | ABT-737 (μM) | Fractional inhibition | CI | Fractional inhibition | CI |
|  |  |  |  |  |  |
| (D)_1_ |  |  |  | ND |  |
| 1 |  | 0.13 |  |  |  |
| 10 |  | 0.35 |  |  |  |
| 100 |  | 0.51 |  |  |  |
| 1000 |  | 0.55 |  |  |  |
|  |  |  |  |  |  |
|  | (D)_2_ |  |  | ND |  |
|  | 0.1 | 0.05 |  |  |  |
|  | 1 | 0.06 |  |  |  |
|  | 10 | 0.18 |  |  |  |
|  | 20 | 0.49 |  |  |  |
|  |  |  |  |  |  |
| (D)_1_ + (D)_2_ |  |  |  | ND |  |
| 1 | 0.1 | 0.15 | 1.640 |  |  |
| 10 | 1 | 0.47 | 0.093 |  |  |
| 100 | 10 | 0.86 | 0.004 |  |  |
